# Molecular Characterization and Mechanistic Insights of a Thermostable Neoagarobiose Hydrolase Aga2457 from *Alteromonas* sp.

**DOI:** 10.3390/md24040123

**Published:** 2026-03-25

**Authors:** Jiang Li, Xinning Pan, Long Chen, Qian Zhang, Zhiyan Wang, Dewi Seswita Zilda, Zhou Zheng

**Affiliations:** 1 Marine Bioresource and Environment Research Center, First Institute of Oceanography, Ministry of Natural Resources, Qingdao 266061, China; 2 Research Center for Applied Microbiology, Research Organization for Life Sciences and Environment, National Research and Innovation Agency Republic of Indonesia, Jakarta Pusat 10340, Indonesia

**Keywords:** neoagarobiose hydrolase, thermostability, molecular dynamics simulation, site-directed mutagenesis, GH117 family

## Abstract

The enzymatic valorization of agarose, a major polysaccharide in red algae, is critical for its application in the food, pharmaceutical, and biotechnology industries. In this study, a gene encoding a thermostable α-neoagarobiose hydrolase, *aga2457*, was cloned from an epiphytic bacterium associated with Indonesian macroalgae. Unlike typical mesophilic GH117 enzymes, recombinant Aga2457 displayed a higher optimal temperature at 50 °C and retained 55% activity after 12 days of incubation at 50 °C. The enzyme specifically hydrolyzes neoagarobiose into D-galactose and 3,6-anhydro-L-galactose, thereby facilitating the complete depolymerization of agarose. Combined molecular dynamics (MD) simulations and site-directed mutagenesis revealed that residues P253, N256, and Q285 are pivotal for substrate recognition and active site stability. These findings highlight Aga2457 as a robust biocatalyst for industrial agar processing and provide structural insights for the rational design of thermostable agarolytic enzymes.

## 1. Introduction

Marine macroalgae represent an abundant and underexploited renewable resource [1]. Compared with terrestrial lignocellulosic materials, algae possess distinct industrial advantages, including rapid growth cycles and the ability to cultivate without occupying arable land [2,3]. Furthermore, their cell walls are devoid of lignin, which significantly facilitates downstream degradation and conversion processes [4,5]. These characteristics confer broad application potential across the food, pharmaceutical, and biotechnology sectors [6,7,8,9]. Consequently, algal polysaccharides have attracted increasing attention in sustainable development and blue biomanufacturing due to their unique bioactivities and tunable physicochemical properties [10,11,12,13,14].

Agarose, the principal structural polysaccharide of red algal cell walls, consists of repeating disaccharide units of β-D-galactose (D-Gal) and 3,6-anhydro-L-galactose (L-AHG) linked alternately by α-1,3 and β-1,4 glycosidic bonds [15], accounting for more than 30% of the dry weight of red algae [16]. As a natural polymer, agarose is widely utilized in the food and pharmaceutical industries [17]. However, its monomeric components hold even greater value: D-Gal exhibits prebiotic properties [18] and participates in various metabolic processes [8,19], while L-AHG possesses significant bioactivities, such as antioxidant, anti-inflammatory, and skin-whitening properties [20,21], Despite this potential, the complex glycosidic linkages within agarose result in poor solubility and limited bioavailability, restricting its direct utilization in large-scale processes [22]. Efficient enzymatic degradation is therefore essential to recover these high-value bioactive monosaccharides.

Traditional acid hydrolysis of agarose typically requires harsh conditions, generating toxic byproducts and degrading sensitive compounds. In contrast, enzymatic hydrolysis provides a green and sustainable alternative [23], offering higher specificity, milder reaction conditions, and reduced environmental impact. This biocatalytic approach avoids the generation of hazardous chemical waste, aligning with principles of green manufacturing [24,25]. The development of efficient agarose-degrading enzymes is thus critical for the sustainable valorization of marine biomass [26].

Currently identified agarose-degrading enzymes are classified into two groups based on their hydrolysis sites: α-agarases (EC 3.2.1.158) and β-agarases (EC 3.2.1.81) [27,28]. α-agarases hydrolyze α-1,3 bonds and are classified into GH96 [29], whereas β-agarases hydrolyze β-1,4 bonds to produce neoagarooligosaccharides (NAOs) and belong to GH16, GH50, GH86, and GH118 families [27]. However, the α-neoagarobiose hydrolase (NABH) belongs to the GH117 family and is indispensable for complete enzymatic hydrolysis of agarose, as it hydrolyzes α-1,3 bonds of neoagarobiose (NA2) from the non-reducing end and produces D-Gal and L-AHG [30]. Monosaccharides possess low molecular weight, excellent water solubility, and good biocompatibility, which make them more versatile and widely applicable in industrial development compared to polysaccharides. Over the past few years, several NABHs have been identified and characterized [31], which potentially promote the comprehensive utilization of algal biomass and overcome obstacles to industrial applications [32]. However, most reported NABHs lack thermostability, a crucial trait for industrial processes where elevated temperatures are required to reduce substrate viscosity and prevent contamination.

To address these process limitations, we cloned and characterized a thermostable α-neoagarobiose hydrolase (NABH), *aga2457*, from a highly active agar-degrading bacterium *Alteromonas* sp. Aga1552 was isolated from the surface of Indonesian macroalgae. The whole-genome sequencing of *Alteromonas* sp. Aga1552 revealed the annotation of 145 polysaccharide-degrading enzyme genes and 16 genes related to agar degradation, including those in the GH16, GH50, and GH117 families [33]. This study evaluates the potential of Aga2457 as a biocatalyst for the efficient production of D-Gal and L-AHG. Unlike its mesophilic counterparts, Aga2457 exhibited superior thermal stability, retaining high catalytic activity at elevated temperatures, which suggests its suitability for industrial applications under harsh operating conditions. Beyond biochemical characterization, homology modeling, molecular docking, and site-directed mutagenesis were employed to elucidate the structural basis of substrate recognition and stability. These findings provide not only a robust enzymatic tool for red algal polysaccharide degradation but also molecular insights to guide future rational engineering for marine biomass biorefinery.

## 2. Results and Discussion

### 2.1. Sequence and Phylogenetic Analysis of Aga2457

The *aga2457* gene consists of 1077 bp and encodes a protein of 358 amino acids (Figure S1), with a calculated molecular mass of 40.48 kDa. No signal peptide was detected, suggesting that *aga2457* is an intracellular protein. Multiple sequence alignment with six representative GH117 family members retrieved from the NCBI database revealed high sequence conservation, particularly in the N-terminal region (Figure S2). Several conserved motifs characteristic of the GH117 family were identified, including KGSFDSHKVHDPCLA (residues 192–205), GGREIKHGVA (228–237), NPISNSGHEVVVVWN (252–265), and TTDGPEKNTIQFA (275–287).

**Figure 1:**
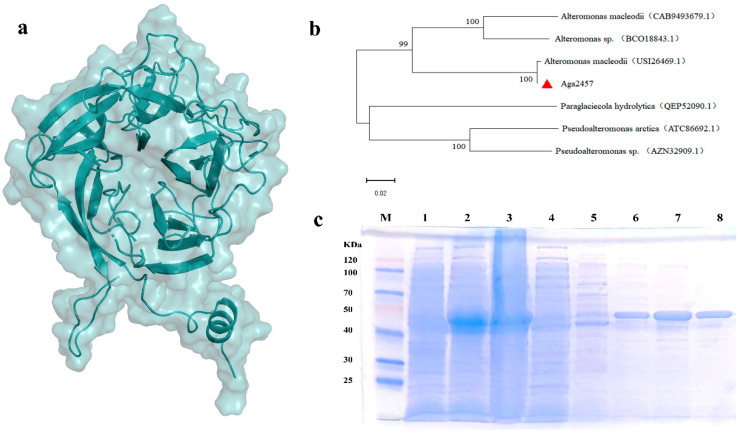
(**a**) Predicted 3D structure of Aga2457. (**b**) Phylogenetic analysis of Aga2457. The red triangle indicates the position of Aga2457 in the phylogenetic tree. (**c**) SDS-PAGE analysis of the recombinant enzyme Aga2457. Note: M: protein marker; Lane 1: total proteins of recombinant *E. coli* BL21 (DE3) not induced with IPTG; Lane 2: total proteins of recombinant *E. coli* BL21 (DE3) induced with IPTG; Lane 3: supernatant of disrupted cells from recombinant *E. coli* BL21 (DE3) induced with IPTG; Lane 4: product eluted with 20 mM imidazole elution buffer; Lane 5: product eluted with 40 mM imidazole elution buffer; Lane 6: product eluted with 80 mM imidazole elution buffer; Lane 7: product eluted with 120 mM imidazole elution buffer; Lane 8: product eluted with 160 mM imidazole elution buffer.

Notably, *aga2457* contains the SxAxxR signature motif within a predicted N-terminal helix–turn–helix (HTH) domain. This structural feature is known to mediate the dimerization required for the oligomerization and function of GH117 enzymes [34,35,36]. Phylogenetic analysis confirmed that *aga2457* clusters tightly with other GH117 family members (Figure 1b), suggesting a common evolutionary origin and conserved catalytic properties.

### 2.2. Expression and Purification of Aga2457

Recombinant Aga2457 was successfully expressed as a soluble protein in *E. coli* BL21 (DE3). The protein was purified to homogeneity using Ni-NTA affinity chromatography. Impurity proteins were removed using low concentrations of imidazole, and the target protein was eluted using 160 mM imidazole. SDS-PAGE analysis revealed a single prominent band with an apparent molecular weight of approximately 43 kDa (Figure 1c, Table S2). This observed molecular weight aligns with the calculated mass of the Aga2457 protein (40.5 kDa) combined with the vector-derived His-tag.

### 2.3. Biochemical Characterization of Aga2457

#### 2.3.1. Effect of Temperature on Activity and Stability

**Figure 2:**
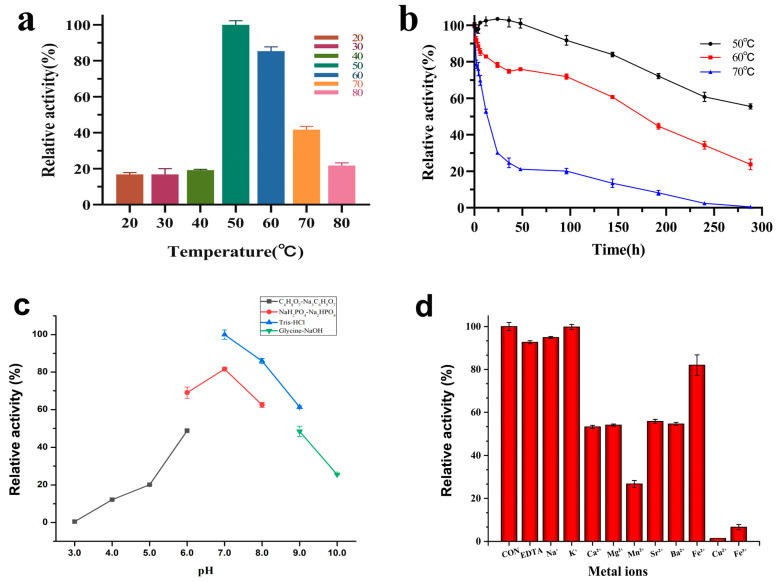
Biochemical properties of Aga2457. Note: (**a**) Relative enzyme activity of recombinase Aga2457 at different temperatures. (**b**) Thermal stability diagram of recombinant enzyme Aga2457. (**c**) Relative enzyme activity of recombinant Aga2457 at different pH levels. (**d**) Relative enzyme activity of recombinant Aga2457 under the action of different metal ions.

The effect of temperature on the enzymatic activity of Aga2457 is shown in Figure 2a. The enzyme exhibited maximal activity at 50 °C, which is notably higher than that of other characterized GH117 family members, such as AgaWH117 (30 °C) and BpGH117 (35 °C) (Table 1) [34,35,36,37,38,39,40,41,42,43,44,45,46,47,48]. At lower temperatures (20–30 °C), the enzyme displayed relatively low activity (17% of maximum). Activity increased sharply with temperature, peaking at 50 °C, and remained high at 60 °C (85.3% relative activity).

The thermostability of Aga2457 was evaluated by monitoring residual activity over an extended incubation period (Figure 2b). Aga2457 exhibited remarkable stability at its optimal temperature (50 °C), retaining nearly 100% activity after 48 h and approximately 55% activity even after 12 days (288 h). At 60 °C, the enzyme remained stable for the first 2 h (>90% retention) and retained 75% activity after 48 h, followed by a gradual decrease to 25% after 12 days. Even at 70 °C, Aga2457 maintained over 50% of its initial activity after 12 h of incubation.

Collectively, these results indicate that Aga2457 possesses a higher thermal optimum and superior stability compared to typical α-neoagarobiose hydrolases, which generally function between 30 and 42 °C. Such thermal resilience suggests that Aga2457 forms a rigid conformation resistant to heat-induced denaturation, making it highly advantageous for industrial bioprocessing. Catalytic reactions at higher temperatures can reduce the risk of bacterial contamination, decrease process cost where cooling may be difficult, increase substrate solubility, and facilitate substrate transformation [49].

#### 2.3.2. Effect of pH on Activity

The pH–activity profile of Aga2457 is illustrated in Figure 2c. The enzyme exhibited optimal activity at pH 7.0 in Tris–HCl buffer. High relative activity was maintained in the neutral-to-slightly alkaline range: the enzyme retained more than 50% of its maximum activity between pH 6.0 (phosphate buffer) to pH 9.0 (Tris–HCl). Conversely, catalytic activity dropped sharply under acidic conditions, with negligible activity observed below pH 5.0. In the alkaline range, activity declined progressively to 44% at pH 9.0 and 20% at pH 10.0.

Notably, the buffer composition significantly influenced enzymatic performance. At equivalent pH values (pH 7.0 and 8.0), Aga2457 exhibited higher activity in Tris–HCl compared to phosphate buffer (PBS), identifying Tris–HCl as the optimal buffer system for this enzyme.

#### 2.3.3. Effect of Metal Ions on the Activity of Aga2457

The influence of various metal ions and chemical reagents on the enzymatic activity of Aga2457 is illustrated in Figure 2d. The enzyme retained high relative activity in the presence of Na^+^, K^+^, and the chelating agent EDTA. The lack of inhibition by EDTA suggests that Aga2457 acts as a metal-independent glycoside hydrolase and does not require divalent cations for catalysis. In contrast, most divalent cations exhibited inhibitory effects to varying degrees. Fe^2+^ caused mild inhibition, with the enzyme retaining approximately 80% of its initial activity. Moderate inhibition was observed with Ca^2+^, Mg^2+^, Sr^2+^, and Ba^2+^, which reduced enzymatic activity to roughly 50%. Notably, Cu^2+^ and Fe^3+^ acted as potent inhibitors, suppressing activity to below 10%. In particular, Cu^2+^ almost completely abolished the catalytic function. This strong inhibition suggests that heavy metal ions may interact with thiol groups or catalytic histidine residues, causing conformational changes or aggregation. No metal ions were found to significantly activate the enzyme.

#### 2.3.4. Enzymatic Kinetic Parameters

**Figure 3:**
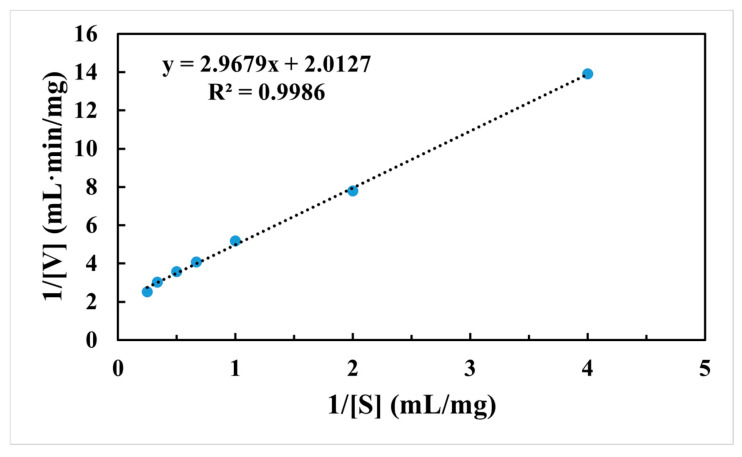
Kinetic parameters of Aga2457.

The values of *K*_m_, *V*_max_ and *k*_cat_ are the most important enzymatic kinetic parameters. After purification, the specific activity of Aga2457 was 451 U/mg, while the *V*_max_, *K*_m_ and *k*_cat_ values were 4.56 mM, 15.24 U/mg and 83.33 S^−1^, respectively (Figure 3). The *K*_m_ of Aga245 was lower than that of some α-neoagarobioses/neoagarooligosaccharides, such as *Bp*GH117 (30.22 mM), Ahg558 (8.01 mM), ScJC117 (11.57 mM), WU-0601 (5.8 mM) and OA-2007 (6.0 mM), but higher than that of Ahg786 (4.5 mM), AhgI (1.03 mM), and *Sd*NABH (3.5 mM) (Table 1) [34,35,36,37,38,39,40,41,42,43,44,45,46,47,48]. The lower the *K*_m_ value for an enzyme, the higher its affinity for the given substrate. Therefore, the low *K*_m_ of Aga245 is indicative of a high affinity towards its substrate.

### 2.4. Analysis of Enzymatic Hydrolysis Products

**Figure 4:**
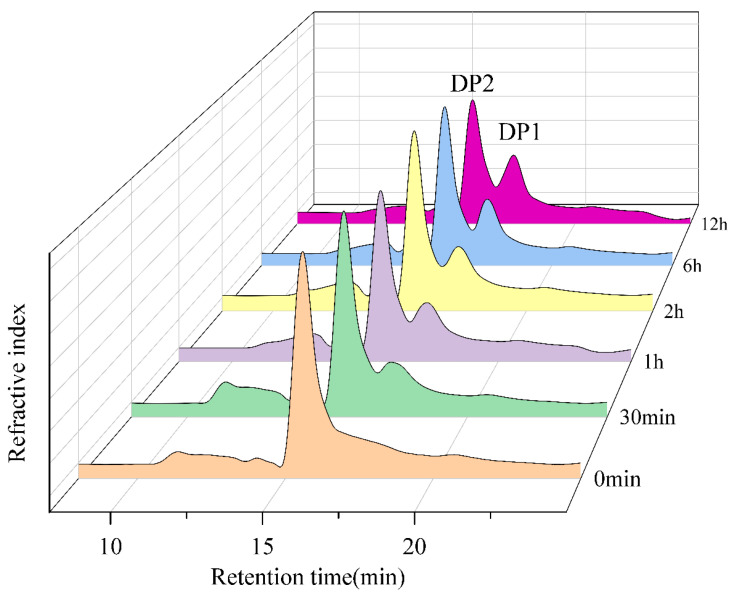
HPLC of hydrolysis products produced by Aga2457. Note: DP2 corresponds to the peak of the standard sample of neoagarobiose; DP1 corresponds to the peak of the standard sample of D-galactose.

To elucidate the mode of action of Aga2457, the hydrolysis products of neoagarobiose were monitored by HPLC at various time intervals (Figure 4). The chromatograms reveal rapid substrate consumption during the initial phase of the reaction. As the reaction proceeded, the peak corresponding to neoagarobiose progressively decreased. Concurrently, peaks corresponding to the constituent monosaccharides, D-Gal and L-AHG, accumulated. These results confirm that Aga2457 functions as a specific α-neoagarobiose hydrolase (NABH). It efficiently cleaves the α-1,3-glycosidic linkage in neoagarobiose to release D-Gal and L-AHG, thereby completing the final step of agarose saccharification.

### 2.5. Structural Modeling and Molecular Docking Analysis

The three-dimensional structure of Aga2457 was predicted using AlphaFold 3. Multiple sequence alignment (MSA) coverage analysis (Figure S4b) revealed a high density of homologous sequences, providing a robust foundation for structural prediction. Five models were generated, all exhibiting highly consistent topologies with a well-conserved core region (Figure S3). Among them, Model_3 displayed the highest confidence, with a mean predicted Local Distance Difference Test (pLDDT) score of 94.8 and a predicted Template Modeling (pTM) score exceeding 0.5 (Table S3). Similarly to the structure of *Sd*NABH, which belongs to the same GH117 family [35], Aga2457 is composed of two parts: an N-terminal extension and a C-terminal five-bladed β-propeller catalytic domain (Figure S3). Consequently, Model_3 was selected for further analysis. The Predicted Alignment Error (PAE) heatmap (Figure S4a) indicated low error values, confirming accurate residue–residue distance predictions. Furthermore, residue-level reliability was corroborated by lDDT analysis (Figure S4c), particularly in the core region (residues 0–330). The stereochemical quality of the predicted structure was validated using Ramachandran plots and VERIFY3D (Figure S5).

**Figure 5:**
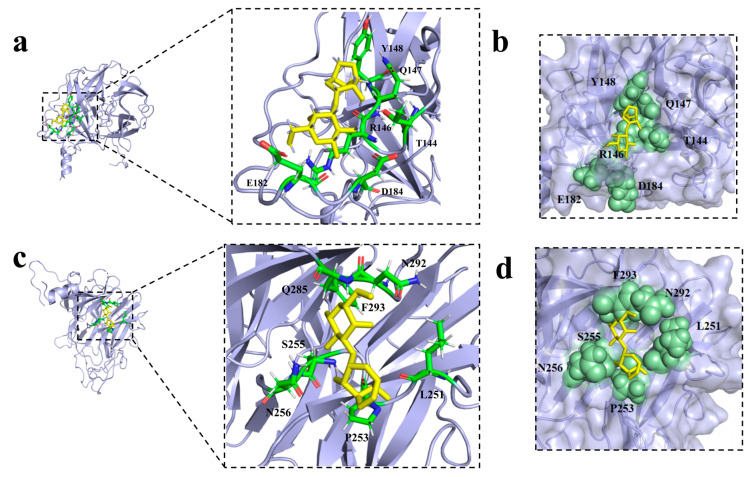
Molecular docking of Aga2457. Note: (**a**,**b**) show schematic diagrams of the binding sites of model 1. (**c**,**d**) show schematic diagrams of the binding sites of model 2.

To elucidate the substrate-binding mechanism, molecular docking was performed with neoagarobiose. The simulation identified two potential substrate-binding clefts located in distinct surface grooves. The two binding modes with the lowest binding energies (indicating the highest affinity) are visualized in Figure 5. Detailed interactions, including hydrogen bonds and hydrophobic contacts involving residues within a 3 Å radius of the ligand, are highlighted in Figure 5a,c. Surface electrostatic potential analysis (Figure 5b,d) further illustrates the spatial accommodation of the substrate within the active pocket. Quantitative data, including binding energies and key interacting residues for the top eight docking poses, are summarized in Table 2 (additional models are presented in Figures S6–S8).

### 2.6. Identification of Key Residues via in Silico Alanine Scanning

To assess the energetic contribution of specific amino acids to substrate binding, in silico alanine scanning was performed on residues located within 3 Å of the ligand. The results, summarized in Table S4, categorize mutations based on their impact on binding energy.

Notably, alanine substitution at nine specific positions—T144, R146, Q147, Y148, W179, K233, P253, N256, and Q285—resulted in a marked decrease in predicted binding affinity (classified as “Destabilizing”). This suggests that the side chains of these residues play pivotal roles in substrate recognition and the stabilization of the enzyme–substrate complex. Furthermore, multiple sequence alignment confirmed that these nine residues are highly conserved across GH117 family enzymes, strongly supporting their functional significance (Figure S2).

### 2.7. Experimental Validation of Key Residues via Site-Directed Mutagenesis

To experimentally validate the roles of the predicted active site residues, nine site-directed mutants were constructed and characterized. The enzymatic activities of these mutants were measured relative to the wild-type (WT) Aga2457 (defined as 100%). As shown in Figure 5a, all mutants exhibited varying degrees of activity loss. Most notably, the mutants P253A, N256A, and Q285A displayed a drastic reduction in catalytic efficiency, with activities retaining only 15.7%, 17.7%, and 14.1% of residual activity, respectively. This significant loss of function underscores the critical role of these three residues in the catalytic mechanism or structural integrity of Aga2457. Although structural and sequence analyses strongly support that the catalytic machinery of the GH117 family comprises Asp97, Asp252 and Glu310 (Zg4663) [36], our result provides insights into the specific contributions of these key residues. Pro253, located in a loop region, likely maintains the rigid β-turn geometry essential for the pocket architecture. Its substitution with alanine presumably destabilizes this local conformation, impeding substrate access. A previous study reported that since the C-terminal loop with an additional β-strand gives a spatial restriction to the substrate binding site, *Sd*NABH, having a cytosolic function, may prefer DP2 to longer NAOSs [35]. Asn256 is predicted to form a hydrogen bond network that stabilizes the enzyme–substrate complex. Disruption of this network by the N256A mutation presumably leads to a marked reduction in substrate binding affinity. Consistent with previous studies on GH117 family enzymes [35,36], Gln285 is highly conserved and is postulated to function as a key catalytic residue, facilitating substrate recognition and proton transfer during the hydrolysis reaction.

**Figure 6:**
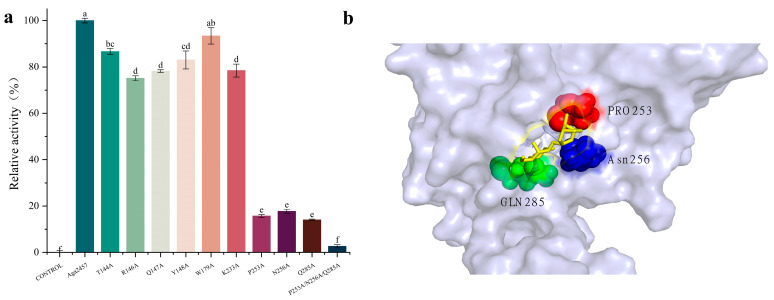
(**a**) Relative enzyme activity of wild-type Aga2457 and various mutants. There are identical letters between two groups, the difference is not significant; there are no identical letters between two groups, the difference is significant. (**b**) Schematic diagram of the binding of three key amino acids to the substrate.

The spatial distribution of these residues highlights their proximity to the catalytic center (Figure 6b). Furthermore, the combinatorial mutant (P253A/N256A/Q285A) exhibited negligible enzymatic activity, confirming the synergistic necessity of these sites for catalysis. In contrast, mutations at the other six positions (T144, R146, Q147, Y148, W179, and K233) resulted in only minor reductions in activity (retaining 75.0–93.4% of WT levels). These residues likely play auxiliary roles, influencing enzyme performance through subtle conformational or electrostatic effects rather than participating directly in the catalytic event.

### 2.8. Molecular Dynamics Simulation Analysis of WT and Triple Mutant

To investigate the structural stability and dynamic behavior of the enzyme–substrate complex, molecular dynamics (MD) simulations were performed for both the wild-type (WT) Aga2457 and the triple mutant (P253A/N256A/Q285A) in complex with neoagarobiose.

#### 2.8.1. RMSD Analysis

**Figure 7:**
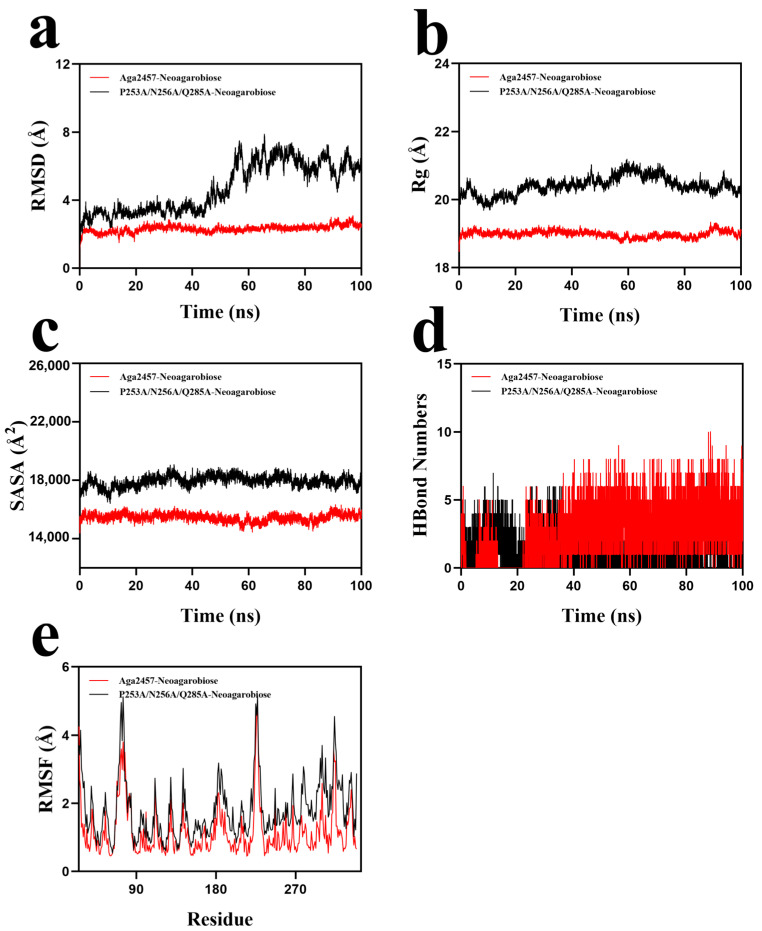
Molecular dynamics simulation of the Aga2457–neoagarobiose and the mutant P253A/N256A/Q285A–neoagarobiose. Note: (**a**) RMSD values of the protein–ligand complex over time. (**b**) Rg values of the protein–ligand complex over time. (**c**) SASA values of the protein–ligand complex over time. (**d**) HBonds values of the protein–ligand complex over time. (**e**) RMSF values of the protein–ligand complex.

RMSD represents the relative positional shifts of the protein structure during the simulations, as well as the overall level of fluctuations in the protein conformation on the basis of the initial structure [50]. The backbone RMSD profiles (Figure 7a) showed that the WT Aga2457 complex reached equilibrium within ~5 ns, maintaining a stable trajectory (average RMSD ~2.5 Å). This indicates a rigid and stable conformation upon substrate binding. In contrast, the triple mutant complex exhibited significantly higher RMSD values and larger fluctuations, failing to reach a stable equilibrium comparable to that of the WT. This increased structural deviation suggests that the simultaneous mutation of P253, N256, and Q285 disrupts critical interactions within the catalytic pocket. The loss of specific side-chain contacts likely weakens the enzyme–substrate affinity and destabilizes the local active site geometry. Lower RMSD values correspond to more stable structures and smaller molecular conformational change [51]. These MD results corroborate the experimental findings, confirming that these residues are essential for maintaining the structural integrity required for substrate binding and catalysis.

#### 2.8.2. Radius of Gyration Analysis

Rg is defined as the average distance from a collection of atoms to their common center of mass, reflecting the structural compactness of the protein [52]. To assess structural compactness, the radius of gyration (Rg) was analyzed. As shown in Figure 7b, the WT complex maintained a consistently lower Rg compared to the triple mutant, confirming that the enzyme retains a tightly folded conformation during catalysis. This expansion in the radius of gyration suggests that the alanine substitutions lead to a relaxation of the protein structure or partial loss of compactness. These results further support the conclusion that the simultaneous mutation of residues P253, N256, and Q285 compromises the overall structural integrity of the enzyme–substrate complex, thereby impairing its catalytic function.

#### 2.8.3. Solvent-Accessible Surface Area Analysis

Solvent-accessible surface area (SASA) analysis (Figure 7c) revealed that the triple mutant exhibited notably higher values than the WT. This increase indicates that the mutations lead to a more open conformation, allowing greater solvent penetration into the substrate-binding pocket.

The expansion of the surface area suggests that the replacement of key residues (P253, N256, Q285) with alanine disrupts the tight packing required for substrate sequestration. Conversely, the wild-type complex maintained a lower and stable SASA profile throughout the simulation, reflecting a compact structure where the ligand is properly encapsulated within the active site, facilitating efficient catalysis.

#### 2.8.4. Hydrogen Bond Analysis

Hydrogen bonds have obvious stability, directionality and relatively high strength [53]. Monitoring of hydrogen bond formation (Figure 7d) indicated that the WT complex maintained an average of ~6 bonds (fluctuating between 0 and 10), reflecting robust substrate anchoring. In contrast, the triple mutant complex exhibited a marked reduction in hydrogen bond formation, with the number of bonds ranging from 0 to 7 and an average of only 3.

This significant decrease confirms that the alanine substitutions at positions P253, N256, and Q285 disrupt the essential hydrogen bonding network required for substrate recognition. The loss of these specific interactions directly correlates with the unstable trajectory (high RMSD) and expanded conformation (high Rg/SASA) observed in the mutant, providing a molecular explanation for the abolished enzymatic activity.

#### 2.8.5. Root Mean Square Fluctuation (RMSF) Analysis

RMSF assesses the overall flexibility of the protein and calculates the standard deviation of all residues of the molecule with respect to the time-averaged coordinates [54]. The larger the RMSF value, the more flexible the corresponding residue. Local residue flexibility was analyzed to pinpoint regions of instability. As shown in Figure 7e, the WT complex exhibited generally lower RMSF values compared to the triple mutant, particularly in the core catalytic domain. The restricted mobility of residues in the WT indicates a rigid framework essential for maintaining active site geometry. In contrast, the P253A/N256A/Q285A mutant displayed significantly higher fluctuations, suggesting that these alanine substitutions induce widespread destabilization of the protein structure.

Collectively, the MD results demonstrate that the WT enzyme maintains a compact, stable conformation underpinned by a robust hydrogen bond network. The P253A/N256A/Q285A mutations disrupt these stabilizing interactions, driving the system toward increased structural flexibility and solvent exposure. These computational findings provide a structural basis for the loss of enzymatic activity, corroborating the experimental observation that residues P253, N256, and Q285 are critical for substrate recognition and catalysis.

## 3. Materials and Methods

### 3.1. Reagents and Materials

The gene encoding the NABH *aga2457* was cloned from *Alteromonas* sp. Aga1552, a highly active agar-degrading bacterium isolated from Indonesian macroalgae. The whole-genome sequencing of *Alteromonas* sp. Aga1552 revealed the annotation of 145 polysaccharide-degrading enzyme genes, including 16 genes related to agar degradation [33]. *Escherichia coli* BL21 (DE3) competent cells and the pET-30a (+) expression vector were obtained from Tiangen (Beijing, China) and Takara (Dalian, China), respectively. DNA markers, protein molecular weight standards, and PCR master mix were supplied by Takara. Kanamycin and isopropyl-β-D-thiogalactoside (IPTG) were sourced from Solarbio (Beijing, China). The neoagarobiose standard was procured from Qingdao BZ Oligo Biotech (Qingdao, China). All other chemicals were of analytical grade. Luria–Bertani (LB) medium was prepared according to standard protocols and sterilized by autoclaving at 121 °C for 20 min.

### 3.2. Gene Synthesis and Sequence Analysis

Physicochemical properties of the putative protein, including theoretical molecular weight and isoelectric point (pI), were predicted using the ExPASy ProtParam tool (http://web.expasy.org/protparam/, accessed on 22 March 2026). Signal peptides and their cleavage sites were identified via the SignalP 4.1 server (http://www.cbs.dtu.dk/services/SignalP-4.1/, accessed on 22 March 2026), while transmembrane helices were analyzed using TMHMM2.0 (http://www.cbs.dtu.dk/services/TMHMM-2.0/, accessed on 22 March 2026). Protein domain architecture was determined using the SMART database (http://smart.embl.de/, accessed on 22 March 2026). To identify homologs, BLASTp 2.15.0+ searches were performed against the NCBI non-redundant database (http://blast.ncbi.nlm.nih.gov/, accessed on 22 March 2026). Multiple sequence alignments were conducted using ClustalW2.1 (https://www.clustal.org/, accessed on 22 March 2026), and secondary structure elements were visualized using ESPript 3.0 (https://espript.ibcp.fr/ESPript/ESPript/, accessed on 22 March 2026). Finally, a phylogenetic tree based on Aga2457 and related sequences was constructed using the Neighbor-Joining method in MEGA 6.0.

### 3.3. Heterologous Expression and Purification of Recombinant Aga2457

The gene encoding *aga2457* with His-tag on the N-terminus was synthesized by GenScript (Nanjing, China) and cloned into the pET-30a (+) vector using *BamHI* and *XbaI* restriction sites. The recombinant plasmid was transformed into *Escherichia coli* BL21 (DE3), and transformants were selected on LB agar supplemented with 50 μg/mL kanamycin and verified by DNA sequencing. For protein expression, the validated strain was cultured in LB broth containing 50 μg/mL kanamycin at 37 °C until the optical density at 600 nm reached 0.6–0.8. Expression was induced by the addition of IPTG to a final concentration of 0.5 mM, followed by incubation at 16 °C for 16 h. Cells were harvested by centrifugation (4025× *g*, 15 min, 4 °C), resuspended in lysis buffer, and disrupted by sonication. After centrifugation at 8050× *g* for 15 min, the supernatant was loaded onto a Ni-NTA affinity column and incubated at 4 °C for 2 h. Bound proteins were eluted using a buffer containing an imidazole gradient (20–160 mM) and analyzed via SDS-PAGE. The target eluent was concentrated and exchanged by a 50 mM Tris–HCl buffer (pH 7.4) by ultrafiltration (10 kDa cut-off membrane, Millipore, Billerica, MA, USA) at 4 °C.

### 3.4. Enzyme Activity Assay

The hydrolytic activity of Aga2457 towards neoagarobiose was determined using the 3,5-dinitrosalicylic acid (DNS) method [55]. Briefly, 1 mL of the enzyme solution (100 μg) was mixed with 1 mL of neoagarobiose solution (3 mM) and incubated at 50 °C for 1 h. The reaction was terminated by boiling the mixture for 15 min. A control was prepared by using a heat-inactivated enzyme before the addition of the substrate. After centrifugation at 8050× *g* for 20 min, 1 mL of the supernatant was mixed with 1.5 mL of DNS reagent. The mixture was boiled for 15 min, cooled on ice, and diluted to a final volume of 25 mL with distilled water. The absorbance was measured at 520 nm. All assays were performed in triplicate.

### 3.5. Determination of Kinetic Parameters of Aga2457

The initial reaction rate of Aga2457 (100 µg) toward different concentrations of neoagarobiose substrate (0.25–4.0 mg/mL) was assayed using the standard method described above. The values of the Michaelis–Menten constant (*K*_m_), maximal reaction rate (*V*_max_) and catalytic constant (*k*_cat_) of Aga2457 were calculated from the Lineweaver–Burk equation using GraphPad Prism 8 statistical package (GraphPad Software, Inc, San Diego, CA, USA) [56]. All biochemical characterization assays were performed in triplicate.

### 3.6. Biochemical Characterization of Purified Recombinant Aga2457

#### 3.6.1. Effect of Temperature on Aga2457 Activity

The effect of temperature on the enzymatic activity of Aga2457 was evaluated in Tris–HCl buffer (pH 7.75 at 25 °C, 7.0 at 50 °C). The reaction mixture, consisting of 1 mL (100 μg) of enzyme solution and 1 mL of neoagarobiose substrate (3 mM), was incubated at temperatures ranging from 20 to 80 °C (at 10 °C intervals) for 1 h. Enzyme activity was quantified using the DNS method. All assays were performed in triplicate. The maximum activity observed was defined as 100%, and relative activities were calculated accordingly.

#### 3.6.2. Thermal Stability of the Recombinant Aga2457

To investigate thermal stability, the enzyme solution was incubated at 50, 60, and 70 °C for varying durations ranging from 0 h to 12 days. After the specified incubation periods, 1 mL of neoagarobiose (3 mM) was added to the enzyme solution, and the reaction was conducted for 1 h at the corresponding incubation temperature. Residual enzyme activity was quantified using the DNS method described above. The activity measured at 0 h was defined as 100%, and relative activities were calculated accordingly.

#### 3.6.3. Effect of pH on the Activity of Recombinant Aga2457

The effect of pH on the enzymatic activity was determined at 50 °C using buffers ranging from pH 3.0 to 10.0. The following buffer systems were used: citric acid–sodium citrate (0.1 M, pH 3.0–6.0), sodium phosphate (0.1 M, pH 6.0–8.0), Tris–HCl (50 mM, pH 7.75–9.78 at 25 °C, 7.0–9.0 at 50 °C), and glycine–NaOH (50 mM, pH 9.0–10.0). Both the enzyme solution and the neoagarobiose substrate were prepared in the respective buffers. The reaction was initiated by mixing 1 mL of enzyme with 1 mL of substrate and incubating for 1 h. Enzyme activity was quantified using the DNS method. The maximum activity was defined as 100%, and relative activities were calculated accordingly.

#### 3.6.4. The Effect of Different Metal Ions on the Activity of Recombinant Aga2457

The influence of various metal ions and EDTA on enzymatic activity was investigated in Tris–HCl buffer (pH 7.0 at 50 °C). The reaction mixture consisted of enzyme solution (100 mg/mL), neoagarobiose (3 mM), and the test additive at a final concentration of 5 mM. The additives tested included NaCl, FeCl_3_, CuCl_2_, MnCl_2_, MgCl_2_, FeCl_2_, CaCl_2_, KCl, SrCl_2_, NiCl_2_, or BaCl_2_, and EDTA. Reactions were incubated at 50 °C for 1 h, and activity was determined using the DNS method. A reaction performed under identical conditions without any additives served as the control (defined as 100%). All assays were performed in triplicate.

### 3.7. Analysis of Hydrolysis Products

The hydrolysis products of neoagarobiose were analyzed using high-performance liquid chromatography (HPLC). The reaction mixture, containing equal volumes of Aga2457 enzyme solution and 3 mM neoagarobiose in Tris–HCl buffer (pH 7.0 at 50 °C), was incubated at 50 °C. Samples were collected at different time intervals (30 min, 1 h, 2 h, 6 h, and 12 h). The reactions were terminated by boiling for 15 min, followed by centrifugation at 8050× *g* for 20 min. The resulting supernatants were lyophilized and redissolved in 5 mg/mL Na_2_SO_4_ solution. The prepared samples were filtered through a 0.22 μm membrane and analyzed using an Agilent HPLC system (Agilent Technologies, Santa Clara, CA, USA) equipped with a refractive index detector (RID) and a TSKgel G2500PWXL column (7.8 mm × 30 cm, 7 μm) maintained at 35 °C. The mobile phase was 5 mg/mL Na_2_SO_4_ supplied at a flow rate of 0.5 mL/min. The injection volume was 20 μL.

### 3.8. Molecular Modeling and Docking Analysis

The three-dimensional (3D) structure of Aga2457 was predicted using the AlphaFold 3 server (https://alphafoldserver.com/, accessed on 22 March 2026). Model quality was assessed based on the predicted Local Distance Difference Test (pLDDT) score, Predicted Alignment Error (PAE) heatmap, predicted Template Modeling (pTM) score, and multiple sequence alignment (MSA) coverage. The model exhibiting the highest overall confidence was selected and further validated using PROCHECK3.5.4 and VERIFY3D via the SAVES v6.0 server (https://saves.mbi.ucla.edu/, accessed on 22 March 2026) to ensure stereochemical consistency and 3D–1D structural compatibility.

For molecular docking, the structure of neoagarobiose (PubChem CID: 164618) was retrieved from the PubChem database. Docking simulations were performed using AutoDock Vina 1.2.2 (The Scripps Research Institute, La Jolla, CA, USA). The binding pose with the lowest affinity energy was selected as the optimal complex. Structural interactions were visualized and analyzed using PyMOL3.1.7.2 (Schrödinger, LLC, New York, NY, USA).

### 3.9. Prediction of Active Sites via in Silico Alanine Scanning

The top eight docking conformations obtained from AutoDock Vina were imported into Discovery Studio 2019 Client (BIOVIA, San Diego, CA, USA) for further analysis. Residues located within a 3 Å radius of the receptor–ligand interface were identified as potential interacting sites. To evaluate the contribution of these specific amino acids to substrate binding, in silico alanine scanning mutagenesis was performed. The effect of each single-point mutation on the binding affinity was evaluated by calculating the mutation energy (ΔΔG_bind_). A positive mutation energy (ΔΔG_bind_ > 0.5 kcal/mol) indicated that the mutation destabilized the enzyme–substrate complex, thereby reducing binding affinity. Conversely, a negative value (<−0.5 kcal/mol) suggested enhanced affinity, while values between −0.5 and 0.5 kcal/mol were considered to have a negligible effect. Residues causing significant destabilization upon mutation to alanine were identified as putative key residues for catalysis or substrate binding.

### 3.10. Site-Directed Mutagenesis and Enzymatic Activity Assay

Site-directed mutagenesis was performed to identify key catalytic or binding residues of Aga2457 using the Fast MultiSite Mutagenesis System (TransGen Biotech, Beijing, China). The recombinant plasmid pET-30a (+)-Aga2457 served as the template. The PCR amplification profile was as follows: pre-denaturation at 95 °C for 3 min, followed by 25 cycles of denaturation at 95 °C for 20 s, annealing at 58 °C for 20 s, and extension at 72 °C for 90 s. The PCR products were verified by 1% agarose gel electrophoresis. A total of 10 μL of the PCR product was digested with DpnI (DMT enzyme, TransGen) to remove the methylated parental plasmid at 37 °C for 1 h. The reaction products were then transformed into competent *E. coli* BL21 (DE3) cells. All mutagenic primers used in this study are listed in Supplementary Table S1.

Expression and purification of the mutant proteins were carried out following the procedures described in Section 2.3. Enzymatic activity was determined using the DNS method (Section 2.4). The wild-type (WT) Aga2457 served as the control (defined as 100% activity). All measurements were performed in triplicate. Based on the initial screening, residues showing a significant reduction in activity were selected for double or multi-point mutagenesis to assess cumulative effects. The structural changes in the mutants were visualized using PyMOL 2.4 to correlate the experimental data with the docking predictions.

### 3.11. Molecular Dynamics Simulation

Molecular dynamics (MD) simulations were performed using the GROMACS 2022 package. The topology for the receptor protein was generated using the CHARMM36 force field [57]. The system was neutralized by adding appropriate counterions using the gmx genion tool. Long-range electrostatic interactions were calculated using the Particle Mesh Ewald (PME) method with a cutoff of 1.0 nm. Covalent bonds were constrained using the SHAKE algorithm, and integration was performed using the Verlet leapfrog algorithm with a time step of 1 fs. Prior to the production run, the system was subjected to energy minimization to remove steric clashes. This involved a sequential protocol: 3000 steps of steepest descent followed by 2000 steps of conjugate gradient optimization. Restraints were applied initially to the solute to relax the solvent, followed by optimization with constrained counterions, and finally, a minimization without constraints. The equilibrated system then underwent a 100 ns production simulation under NPT ensemble conditions at 310 K and 1 bar. Trajectory analysis was performed using standard GROMACS utilities, including gmx rms for root-mean-square deviation (RMSD), gmx rmsf for root-mean-square fluctuation (RMSF), gmx hbond for hydrogen bond monitoring, gmx gyrate for radius of gyration (Rg), and gmx sasa for solvent-accessible surface area (SASA).

### 3.12. Statistical Analysis

All data were analyzed using SPSS 27.0. and are presented as means ± standard deviation (SD). Statistical significance was performed using a one-way ANOVA, followed by post hoc Tukey’ s test.

## 4. Conclusions

In this study, Aga2457 was characterized as a thermostable α-neoagarobiose hydrolase belonging to the GH117 family. The enzyme exhibits superior catalytic efficiency and exceptional thermal stability at 50 °C, distinguishing it from typical mesophilic homologues. By integrating structural modeling, molecular dynamics simulations, and site-directed mutagenesis, we elucidated the molecular basis of substrate interaction. Specifically, residues P253, N256, and Q285 were identified as critical determinants for maintaining the structural integrity of the active pocket and facilitating catalysis.

Overall, the robust thermostability and high catalytic efficiency of Aga2457 position it as a promising biocatalyst for the industrial production of D-Gal and L-AHG. Furthermore, the structural insights provided in this work establish a solid framework for the rational design of GH117 enzymes, paving the way for more efficient agarose valorization strategies in marine biotechnology.

## Figures and Tables

**Table 1:** Comparison of biochemical properties with other GH117 family and characterized α-Neoagarobiose/neoagarooligosaccharide hydrolases.

Strain (Enzyme)	Molar Mass of Subunit (kDa)	Location of Protein	Effect of Metal Ion				Optimum		References
			Activation	Inhibition	*K*_m_ (mM)	*V*_max_ (U/mg)	Temp. (°C)	pH	
Aga2457	40.48	Cytosolic	n.a.	Ba^2+^, Ca^2+^, Mn^2+^ Cu^2+^, Fe^3+^, Mg^2+^	4.56	15.42	50	7.0	This study
*Bacteroides plebeius* (*Bp*GH117)	45.6	Extracellular	n.a.	n.a.	30.22	54.84	35	9.0	[37]
*Gayadomonas joobiniege G7* (Ahg558)	40.8	n.a.	Mn^2+^	Cu^2+^, Mg^2+^	8.01	133.33	30	9.0	[38]
*Streptomyces coelicolor* A3(2) (ScJC117)	41	Extracellular	Mg^2+^	Ba^2+^, Ca^2+^, Co^2+^, Fe^3+^, Zn^2+^, Ni^2+^	11.57	n.a.	30	6.0	[39]
*Gayadomonas joobiniege* (Ahg786)	45.18	Extracellular	Mn^2+^	Cu^2+^, Mg^2+^, Zn^2+^, Ni^2+^	4.5	1.33	15	7.0	[40]
*Cellvibrio* sp. WU-0601	42	Cytosolic	Mn^2+^, Mg^2+^	Ag^+^, Hg^2+^, Cu^2+^, Ni^2+^	5.8	60	25	6.0	[41]
*Agarivorans gilvus* WH0801 (AgaWH117)	41	Cytosolic	n.a.	n.a.	6.45	6.98	30	6.0	[42]
*Cellulophaga* sp. W5C (AhgI)	45	Extracellular	Ca^2+^	n.a.	1.03	10.22	20–30	7.0	[34]
*Saccharophagus degradans* 2–40T (SdNABH)	41.6	Cytosolic	n.a.	Zn^2+^, Ni^2+^, Cu^2+^, Co^2+^	3.5	n.a.	42	6.5	[35]
*Zobellia galactanivorans* (AhgA)	41	Extracellular	n.a.	n.a.	n.a.	n.a.	n.a.	n.a.	[36]
*Cellvibrio* sp. OA-2007	40	Cytosolic	n.a.	n.a.	6	19	32	7.0–7.2	[43]
*Vibrio* sp. JT0107	42	Cytosolic	n.a.	n.a.	5.37	92	30	7.7	[44]
*Pseudomonas atlantica*	10	Periplasmic	Na^+^	n.a.	n.a.	n.a.	n.a.	7.3–8.0	[45]
*Bacillus* sp. MK03	42	Extracellular	Mg^2+^	Ag^+^, Ni^2+^, Cu^2+^, Hg^2+^	n.a.	22.2	30	6.1	[46]
*Cytophaga flevensis*	n.a.	Cytosolic	n.a.	Ag^+^, Hg^2+^, Zn^2+^, Pb^2+^	n.a.	n.a.	25	6.75	[47]
*Cellvibrio* sp.GH117A α-NABH	40.9	n.a.	Mn^2+^	n.a.	n.a.	n.a.	35	7.5	[48]

Note: Aga2457 exhibits the highest optimal reaction temperature of 50 degrees Celsius. Further elevation of the temperature to 70 °C and 80 °C resulted in a decline in activity to 41.7% and 21.7%, respectively.

**Table 2:** Binding energy and amino acid residues at the binding site for different dockings.

MODEL	Binding Energy (kcal/mol)	Amino Acids Within 3 Å of the Neoagarobiose
1	−5.63	T144, R146, Q147, Y148, E182, D184
2	−5.524	L251, P253, S255, N256, Q285, N292, F293
3	−5.51	L251, P253, I254, S255, N256, L291, N292, F293
4	−5.493	P142, T144, R146, Q147, Y148, E182, D184
5	−5.43	I254, S255, N256, E280, L291, F293, E294
6	−5.347	P142, T144, R146, Q147, Y148, K175, W179, M221
7	−5.341	T144, R146, Q147, W179, E182, D184
8	−5.31	K233, L251, P253, N256, N292, F293

## Data Availability

All data generated or analyzed during this study are included in this article and its Supporting Information. Additional information is available from the corresponding author upon reasonable request.

## Supplementary Materials

The following supporting information can be downloaded at: https://www.mdpi.com/article/10.3390/md24040123/s1, Table S1: Primers required for mutant construction; Table S2: Purification step of Aga2457; Table S3: Information related to Model_1-5; Table S4: Table showing mutation energy after alanine scanning of different models and their effects; Figure S1: Sequence of Aga2457 gene and its encoded amino acid sequence; Figure S2: Multiple sequence alignment of the Protein encoded by Aga2457; Figure S3: Five prediction models of Aga2457; Figure S4: (a) Five prediction models predict alignment errors. (b) The distribution map of the coverage area of multiple sequence alignments. (c) The local distance difference test evaluates the local accuracy of the predicted structure; Figure S5: The rationality evaluation of Aga2457 three-dimensional structure. Note: (a): Ramachandran plot of the structures of Aga2457; (b): The VERIFY3D results of the structures of Aga2457; Figure S6: Molecular docking of Aga2457 (a) and (b) show schematic diagrams of the binding sites of model 3. (c) and (d) show schematic diagrams of the binding sites of model 4; Figure S7: Molecular docking of Aga2457 (a) and (b) show schematic diagrams of the binding sites of model 5. (c) and (d) show schematic diagrams of the binding sites of model 6; Figure S8: Molecular docking of Aga2457 (a) and (b) show schematic diagrams of the binding sites of model 7. (c) and (d) show schematic diagrams of the binding sites of model 8.
