# Supplementary material for: Molecular Characterization and Mechanistic Insights of a Thermostable Neoagarobiose Hydrolase Aga2457 from Alteromonas sp
Source: Mar Drugs. 2026 Mar 25;24(4):123. doi: 10.3390/md24040123 (PMC13118177; doi:10.3390/md24040123)
Supplement: Supplementary file 1 [file marinedrugs-24-00123-s001.zip › marinedrugs-4220336-supplementary.pdf]

Supplementary Materials

Supplementary Table

Supplementary Table S1. Primers required for mutant construction

Table S1

Gene name	Primers	Oligonucleotide sequence （5’-3’）
T144A	Forward	CGTATGCGAATCGCCAATATGAAGAAATTGCG
	Reverse	TTGGCGATTCGCATACGGCGCCTGCACGCTCT
R146A	Forward	CGTATACCAATGCGCAATATGAAGAAATTGCGATCGC
	Reverse	TTGCGCATTGGTATACGGCGCCTGCACGCTCT
Q147A	Forward	CAATCGCGCGTATGAAGAAATTGCGATCGCGT
	Reverse	CTTCATACGCGCGATTGGTATACGGCGCCTGC
Y148A	Forward	TCGCCAAGCGGAAGAAATTGCGATCGCGTATG
	Reverse	TTTCTTCCGCTTGGCGATTGGTATACGGCGCC
W179A	Forward	AAGATGGCGAAGCGGAAGGCGAAGAAGATAATCGCT
	Reverse	TTCCGCTTCGCCATCTTTCGCCGGGCTAACAA
K233A	Forward	GAAATTGCGCATGGCGTGGCGGTTAGCAATAG
	Reverse	ACGCCATGCGCAATTTCGCGGCCGCCCATATT
P253A	Forward	GCGATCTGAATGCGATTAGCAACAGCGGCCATGA
	Reverse	AATCGCATTCAGATCGCTTTTTTTATACGGAC
N256A	Forward	AATCCGATTAGCGCGAGCGGCCATGAAGTGGTGG
	Reverse	CTCGCGCTAATCGGATTCAGATCGCTTTTTTT
Q285A	Forward	TACCATTGCGTTTGCGGAAGACGGCCTGAATT
	Reverse	CCGCAAACGCAATGGTATTTTTTTCTGGACCATCG
P253A/N256A/Q285A	Forward1	TGAATGCGATTAGCGCGAGCGGCCATGAAGTGGTGG
	Reverse1	TCGCGCTAATCGCATTCAGATCGCTTTTTTTATACGGA
	Forward2	TACCATTGCGTTTGCGGAAGACGGCCTG
	Reverse2	CCGCAAACGCAATGGTATTTTTTTCTGGACCATCG

Supplementary Table S2. Purification step of Aga2457

Table S2

Purification step	Total protein (mg)	Total activity (U)	Specific activity (U/mg)	Purification (fold change)	Yield (%)
Crude enzyme	12.4	3214.6	259	1	100
80 mM imidazole	7.2	2659.4	397	1.53	82.73
120 mM imidazole	5.6	2418.7	432	1.67	75.24
160 mM imidazole	4.5	2028.6	451	1.74	63.11

Supplementary Table S3. Information related to Model_1-5

Table S3

**Model**	**pLDDT**	**pTM**
Model_1	94.6	0.918
Model_2	94.4	0.923
Model_3	94.8	0.93
Model_4	94.8	0.925
Model_5	94.6	0.93

Supplementary Table S4. Table showing mutation energy after alanine scanning of different models and their effects

Table S4

Model	Mutation	Mutation Energy (kcal/mol)	Effect
1	T144>A	0.37	NEUTRAL
	**R146>A**	**1.39**	**DESTABILIZING**
	**Q147>A**	**0.75**	**DESTABILIZING**
	**Y148>A**	**0.66**	**DESTABILIZING**
	G181>A	-0.28	NEUTRAL
	E182>A	0.29	NEUTRAL
	E183>A	0	NEUTRAL
	D184>A	-0.11	NEUTRAL
2	L251>A	0.13	NEUTRAL
	**P253>A**	**0.56**	**DESTABILIZING**
	I254>A	0.14	NEUTRAL
	S255>A	0.01	NEUTRAL
	**N256>A**	**0.51**	**DESTABILIZING**
	N292>A	0.12	NEUTRAL
	F293>A	0.21	NEUTRAL
3	L251>A	0.15	NEUTRAL
	P253>A	0.31	NEUTRAL
	I254>A	0.28	NEUTRAL
	S255>A	0.03	NEUTRAL
	**N256>A**	**0.71**	**DESTABILIZING**
	E280>A	0.23	NEUTRAL
	L291>A	0.05	NEUTRAL
	N292>A	0.18	NEUTRAL
	F293>A	0.21	NEUTRAL
	E294>A	0.07	NEUTRAL
4	P142>A	0.25	NEUTRAL
	**T144>A**	**0.57**	**DESTABILIZING**
	**R146>A**	**1.05**	**DESTABILIZING**
	Q147>A	0.49	NEUTRAL
	Y148>A	0.36	NEUTRAL
	E182>A	0.25	NEUTRAL
	D184>A	0.02	NEUTRAL
5	L251>A	0.3	NEUTRAL
	P253>A	0.13	NEUTRAL
	I254>A	0.21	NEUTRAL
	S255>A	0.03	NEUTRAL

	N256>A	0.44	NEUTRAL
	E280>A	0.06	NEUTRAL
	**Q285>A**	**0.7**	**DESTABILIZING**
	L291>A	0.04	NEUTRAL
	N292>A	0.11	NEUTRAL
	F293>A	0.17	NEUTRAL
	E294>A	0.05	NEUTRAL
6	P142>A	0.37	NEUTRAL
	**T144>A**	**0.72**	**DESTABILIZING**
	R146>A	0.41	NEUTRAL
	**Q147>A**	**0.66**	**DESTABILIZING**
	**Y148>A**	**1.17**	**DESTABILIZING**
	**K175>A**	**0.8**	**DESTABILIZING**
	**W179>A**	**0.63**	**DESTABILIZING**
	**M221>A**	**0.61**	**DESTABILIZING**
7	T144>A	0.36	NEUTRAL
	**R146>A**	**1.42**	**DESTABILIZING**
	Q147>A	0.28	NEUTRAL
	Y148>A	0.44	NEUTRAL
	**W179>A**	**1.04**	**DESTABILIZING**
	E182>A	0.26	NEUTRAL
	D184>A	0.14	NEUTRAL
	M221>A	0.18	NEUTRAL
8	**K233>A**	**0.8**	**DESTABILIZING**
	L251>A	0.14	NEUTRAL
	N252>A	0.01	NEUTRAL
	**P253>A**	**0.98**	**DESTABILIZING**
	I254>A	0.13	NEUTRAL
	**N256>A**	**0.79**	**DESTABILIZING**
	N292>A	-0.1	NEUTRAL
	F293>A	0.15	NEUTRAL

Supplementary Figures

Supplementary **Figure S1.** Sequence of Aga2457 gene and its encoded amino acid sequence

```
1      ATGTCTCAGAGAATAAGTTTAGCGTCGAAACGTGCGATTGAGCGAGGTTACGATAATAAAGGGCCTGAGTGGATGACTGAATTTGATGTTATGCCATTAAAAGGCGATTTTGCTTATCAA
1       M  S  Q  R  I  S  L  A  S  K  R  A  I  E  R  G  Y  D  N  K G  P  E  W  M  T  E  F  D  V  M  P  L  K  G  D  F  A  Y  Q
121    GAAGGCGTTATCCGCAGGGACCCATCATCTGTTATTAAAGTACAGGGTGTATATCATTGTTGGTACACAAAGGGAGAGGGTGAGACCGTCGGTTTCGATTCTGAAAACGAAAACGACAAA
41      E  G  V  I  R  R  D  P  S  S  V  I  K  V  Q  G  V  Y  H  C  W  Y  T  K  G  E  G  E  T  V  G  F  D  S  E  N  E  N  D  K
241    GTTTTTCCGTGGGATTTAACTGAGGTCTGGCATGCCACATCTGAAGATGGAGAAACCTGGAAAGAGCAGGGTGTAGCAATAACTAGAGGTAAAGCTGGAGACTATGATGATAGAGCCGTA
81      V  F  P  W  D  L  T  E  V  W  H  A  T  S  E  D  G  E  T  W  K  E  Q  G  V  A  I  T  R  G  K  A  G  D  Y  D  D  R  A  V
361    TTTACTCCTGAGGTTTTCCATCACGAAGGTAGGTTTTATCTTGTTTATCAAAGTGTACAAGCTCCTTACACCAATAGACAGTATGAAGAGATAGCTATCGCTTATGCTGATAGTCCATTT
121     F  T  P  E  V  F  H  H  E  G  R  F  Y  L  V  Y  Q  S  V  Q  A  P  Y  T  N  R  Q  Y  E  E  I  A  I  A  Y  A  D  S  P  F
481    GGCCCTTGGACTAAGTCGTCGTCACCCGTGGTATCACCTGCTAAAGACGGAGAATGGGAGGGGGAGGAAGATAACCGCTTTAACGTGAAATCTAAGGGGAGCTTCGATAGCCATAAAGTC
161     G  P  W  T  K  S  S  S  P  V  V  S  P  A  K  D  G  E  W  E  G  E  E  D  N  R  F  N  V  K  S  K  G  S  F  D  S  H  K  V
601    CACGACCCATGCTTGTTGTTCTTTAACGACAAATTCTACCTTTATTATAAAGGTGAAACCATGGGCGAATCCATGAACATGGGTGGAAGGGAAATTAAACACGGTGTAGCGGTATCAAAC
201     H  D  P  C  L  L  F  F  N  D  K  F  Y  L  Y  Y  K  G  E  T  M  G  E  S  M  N  M  G  G  R  E  I  K  H  G  V  A  V  S  N
721    TCTCCACTTGGCCCTTATAAAAAATCAGATTTAAATCCTATATCAAATAGTGGTCATGAGGTTGTTGTTTGGAACTACAAAGGCGGAATAGCAACACTATTAACAACAGATGGGCCTGAA
241     S  P  L  G  P  Y  K  K  S  D  L  N  P  I  S  N  S  G  H  E  V  V  V  W  N  Y  K  G  G  I  A  T  L  L  T  T  D  G  P  E
841    AAGAACACGATTCAGTTTGCAGAAGATGGTTTGAATTTCGAAATTATGGCTCACATAAAAGGCGCTCCCGAGGCGATAGGGCTTTTTCGTGAAGGGATTGATGAAACGACGCCACCAGGG
281     K  N  T  I  Q  F  A  E  D  G  L  N  F  E  I  M  A  H  I  K  G  A  P  E  A  I  G  L  F  R  E  G  I  D  E  T  T  P  P  G
961    CTTACCTGGGGATTGTGCCATAAATATGATTCTAGTTGGAACTGGAACTATATTTGTAAATTCAAAACAAGAAAACAAATACTCGACGCTGGTACATTTCAGAATACGGGAAGTTAA
321     L  T  W  G  L  C  H  K  Y  D  S  S  W  N  W  N  Y  I  C  K  F  K  T  R  K  Q  I  L  D  A  G  T  F  Q  N  T  G  S  *
```

Figure S1.

Supplementary Figure S2. Multiple sequence alignment of the Protein encoded by Aga2457

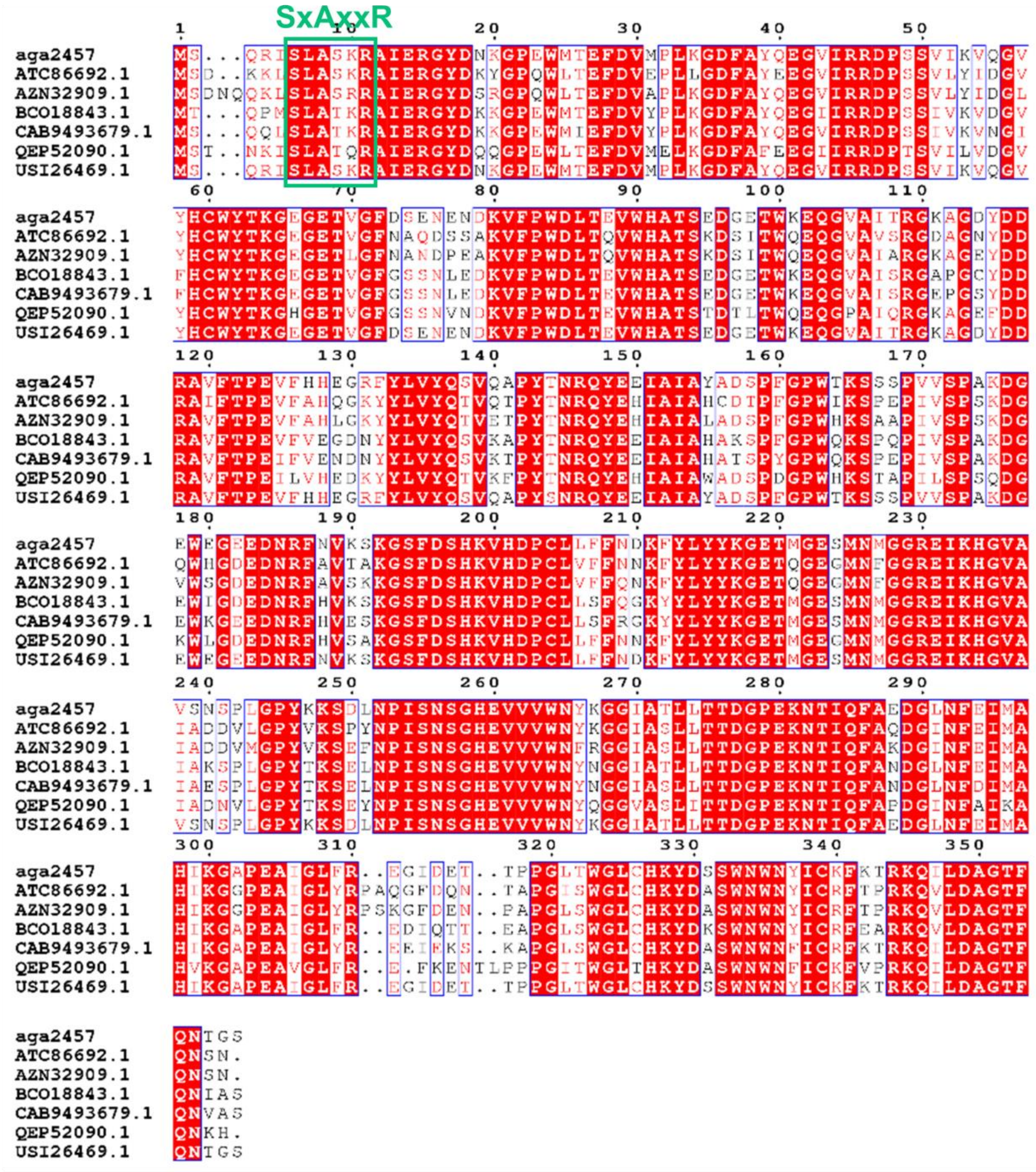

Figure S2.

Supplementary Figure S3. Five prediction models of Aga2457

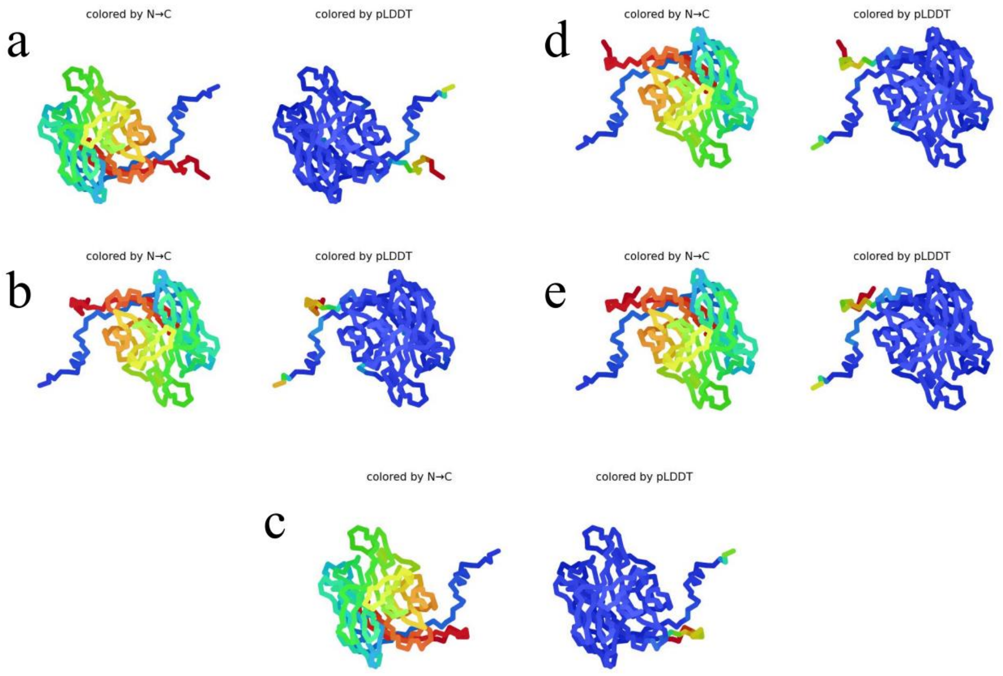

Figure S3.

Supplementary Figure S4. (a) Five prediction models predict alignment errors. (b) The distribution map of the coverage area of multiple sequence alignments. (c) The local distance difference test evaluates the local accuracy of the predicted

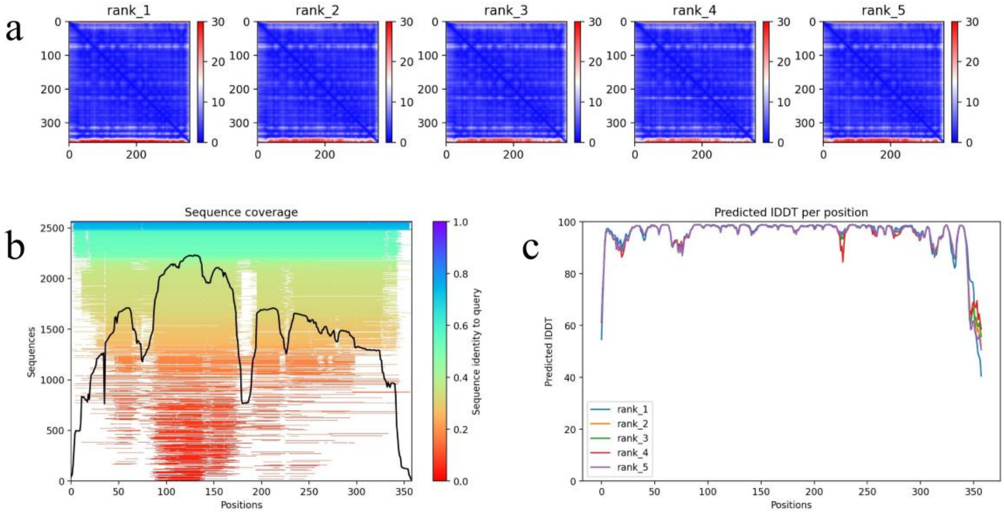

Figure S4.

Supplementary Figure S5. The rationality evaluation of Aga2457 three-dimensional structure. Note: (a): Ramachandran plot of the structures of Aga2457; b: The VERIFY3D results of the structures of Aga2457

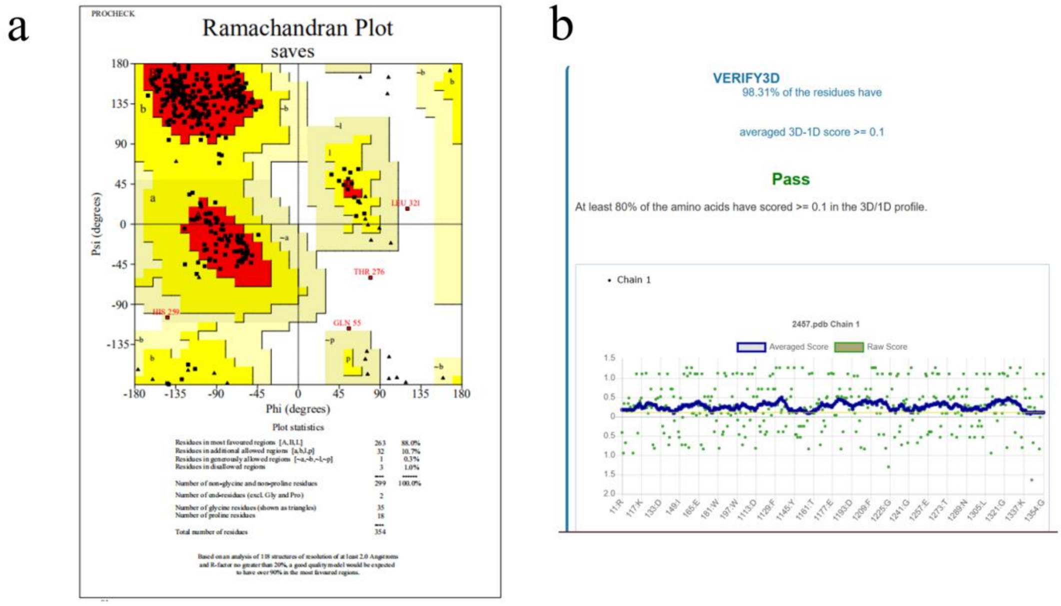

Figure S5

Supplementary Figure S6. Molecular docking of Aga2457 (a) and (b) show schematic diagrams of the binding sites of model 3. (c) and (d) show schematic diagrams of the binding sites of model 4

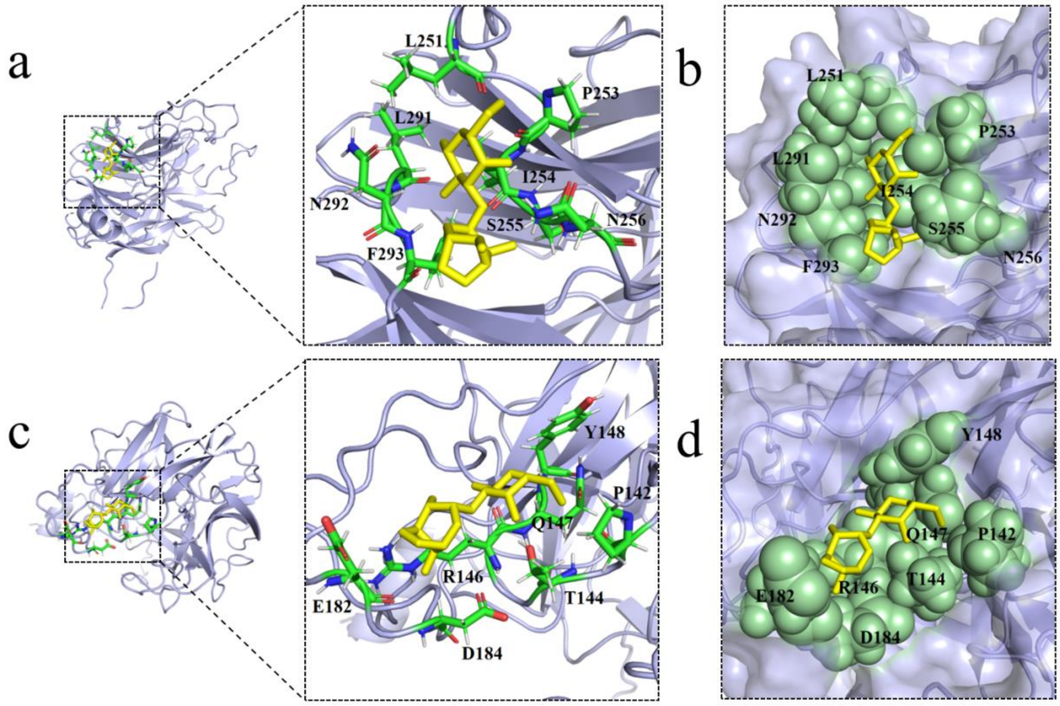

Figure S6

Supplementary Figure S7. Molecular docking of Aga2457 (a) and (b) show schematic diagrams of the binding sites of model 5. (c) and (d) show schematic diagrams of the binding sites of model 6

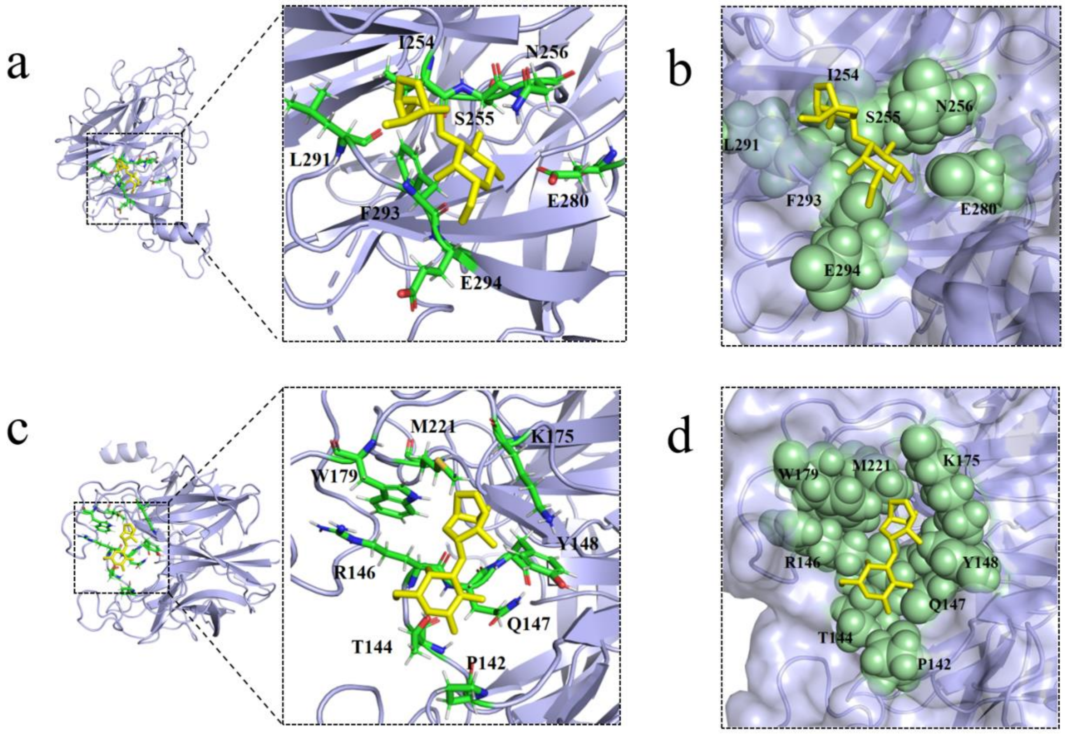

Figure S7

Supplementary Figure S8. Molecular docking of Aga2457 (a) and (b) show schematic diagrams of the binding sites of model 7. (c) and (d) show schematic diagrams of the binding sites of model 8

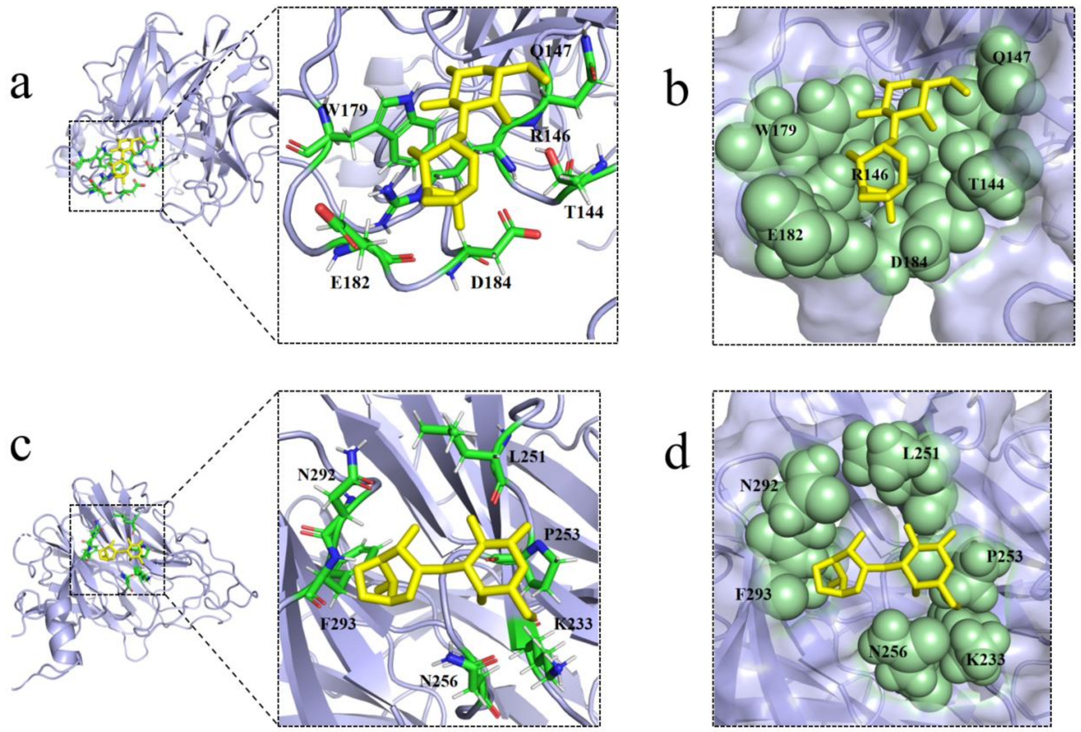

Figure S8
